# HLA-G expression in peritumoral fundic gland mucous neck cells, but not in tumor lesions, related to poor survival in patients with gastric cancer

**DOI:** 10.3389/fimmu.2025.1660054

**Published:** 2025-10-16

**Authors:** Xia Zhang, Qiu-Yue Han, Jian-Gang Zhang, Wei-Hua Yan, Aifen Lin

**Affiliations:** ^1^ Biological Resource Center, Taizhou Hospital of Zhejiang Province, Wenzhou Medical University, Linhai, Zhejiang, China; ^2^ Key Laboratory of HLA-G Research and Development of Taizhou, Taizhou Hospital of Zhejiang Province, Wenzhou Medical University, Linhai, Zhejiang, China; ^3^ Key Laboratory of Minimally Invasive Techniques and Rapid Rehabilitation of Digestive System Tumor of Zhejiang Province, Taizhou Hospital of Zhejiang Province, Wenzhou Medical University, Linhai, Zhejiang, China; ^4^ Medical Research Center, Taizhou Hospital of Zhejiang Province, Wenzhou Medical University, Linhai, Zhejiang, China

**Keywords:** HLA-G, gastric cancer, peritumoral tissue, mucous neck cell, survival

## Abstract

**Introduction:**

The concept that human leukocyte antigen-G (HLA-G) expression is specifically in various malignant lesions but absent in adjacent normal tissues has been well documented, and HLA-G is acknowledged as a novel immune checkpoint.

**Methods:**

In this study, HLA-G expression in 523 gastric cancer (GC) lesions and 283 case-matched peritumoral tissues (PTTs) was analyzed by immunohistochemistry. The clinical significance of HLA-G expression in GC lesions and case-matched PTTs was evaluated, and a specific HLA-G-positive subpopulation in PTTs was identified.

**Results:**

Data showed that HLA-G expression was significantly different between GC lesions (370/523, 70.7%) and PTTs (238/283, 84.1%; *p*<0.001). HLA-G in GC lesions was more frequently observed in older patients (76.2% vs. 65.3%, *p*=0.006), and HLA-G in PTTs was more commonly observed in female patients (92.0% vs. 80.5%, *p*=0.014). Survival analysis revealed that HLA-G expression in PTTs (overall survival: 42.0 months vs. 56.7 months, *p*=0.023), but not in GC lesions (52.1 months vs. 50.9 months, *p*=0.623), is significantly associated with poor prognosis. The fundic gland mucous neck cells were the only subpopulation that expressed HLA-G.

**Discussion:**

Our findings, for the first time, suggest that fundic gland mucous neck cell HLA-G expression in PTTs is associated with GC progression.

## Introduction

Human leukocyte antigen-G (HLA-G) expression was firstly evidenced in cytotrophoblasts in 1990, and that HLA-G rarely expressed in healthy adult tissues was well acknowledged (1, 2). To date, HLA-G expression has been observed only among few healthy tissues such as pancreatic islet β cells, erythroblasts, thymic medullary and subcapsular epithelium, and prostate gland tubule glandular epithelia and in glandular secretions (3–6). In contrast, aberrant HLA-G expression has been repeatedly observed in pathological conditions such as malignant lesions, virus-infected cells, and organ grafts (7–9).

Early studies revealed that HLA-G is an immune suppressor that is important to maintain the fetal–maternal immunotolerance during implantation and pregnancy (10). Regarding cancer, in 1998, it was first found that HLA-G expression is restricted to melanoma lesions, but not in adjacent normal skin tissues, and that HLA-G could inhibit NK cell function to enhance cancer immune evasion (11). Motivated by this finding, researchers evaluated HLA-G expression and its clinical relevance in more than 30 types of different pathological cancers. Most findings revealed that HLA-G expression was significantly associated with disease progression and poor survival of patients with cancer; however, the findings were not consistent among different studies or cancer types (12, 13).Mechanistically, through interactions with immune receptors such as ILT2, ILT4, and KIR2DL4, HLA-G could render multiple-level immune suppression function (14). Briefly, HLA-G could inhibit the cytotoxicity function of NK cell and CD8+ T cells, proliferation of CD4+ T cells, NK cells, and B cells, and differentiation and maturation of B cells and dendritic cells (15). Otherwise, HLA-G could also shift Th1 to Th2 immune response, increase the accumulation of MDSC and Treg, polarize M1 to M2 macrophages, and induce tolerogenic T cells, NK cells, and dendritic cells (14, 16).

Given its tumor-specific expression and immune inhibitory function and its anti-tumor efficacy further solidified by pre-clinical HLA-G-targeted *in vitro* and *in vivo* studies (17–21), HLA-G is expected to act as a promising immune checkpoint. In addition, HLA-G-targeted clinical trials for a wide range of advanced solid cancer immunotherapies have been evaluated since 2020 (https://clinicaltrials.gov/search?cond=HLA-G) (22).

While most previous studies have focused on the cancer itself and aberrant HLA-G expression has been repeatedly observed in various malignant lesions, few studies have reported weak or absent HLA-G expression in peritumoral tissues (PTTs) (6, 23). Whether HLA-G is expressed in PTTs and its clinical importance remain largely unknown. PTTs have distinct transcriptomic, proteomic, and biological behavioral features from the corresponding healthy tissues and cancer lesions (24, 25). Moreover, the clinical significance, such as prognostic and diagnostic values of biomarkers in PTTs, has been highlighted (26, 27).

In this study, HLA-G expression in 523 gastric cancer (GC) lesions and 283 case-matched PTTs was analyzed using immunohistochemistry (IHC), and the clinical significance of HLA-G expression in GC lesions and case-matched PTTs was evaluated. For the first time, we reported that HLA-G expression in PTTs is of obvious significance for the survival of patients with GC, that HLA-G expression is specifically restricted to mucous neck cells in PTTs and GC lesions, and that HLA-G expression in PTT, but not in GC lesions, is related to poor survival of GC patients.

## Materials and methods

### Tissue microarray

GC tissue microarrays (TMAs) were purchased from Shanghai Outdo Biotech Company (Shanghai, China). Patients underwent surgery between February 26, 2002 and September 4, 2009, and none of the patients underwent preoperative radiotherapy, chemotherapy, or other medical interventions. The clinicopathological characteristics of patients with GC, including age, sex, stage, tumor–node–metastasis (TNM) and American Joint Committee on Cancer (AJCC) stage, follow-up data, and survival information, were recorded and archived at the National Engineering Center for Biochip. The disease stage classification of patients with GC was based on the 7th AJCC Cancer Staging System (28). PTT samples were collected >5 cm from the margin of the tumor, and no metastatic specimens were included.

In total, 561 primary GC samples and 289 case-matched PTTs were included in the TMAs. After IHC staining, 38 cases and six PTT samples were detached from the TMA. Ultimately, 523 GC and 283 case-matched PTT samples were included in this study. The median age of the cohort was 63 years (range: 28–89 years). Among the 523 patients with GC (*n*_male_=353, *n*_female_=170), 49, 148, 297, and 25 had AJCC stage I, II, III, and IV, respectively. The latest follow-up was completed in July 2015, at an average follow-up of 40.7 months. Patient overall survival was calculated from the date of surgery to the date of the event or the latest follow-up.

Furthermore, to explore the HLA-G-expressing subpopulation of gastric cells, six PTT samples for Western blot and additional four PTTs and two GC lesions (formalin-fixed paraffin-embedded) for multiplex immunohistochemistry (mIHC) were obtained from the Biological Resource Center, National Human Genetic Resources Platform of China (YCZYPT, 2017), Taizhou Hospital of Zhejiang Province, for IHC and multiplex IHC (mIHC) staining. Written informed consent was obtained from each participant. The study was approved by the Ethics Committee of Shanghai Outdo Biotech Company and the Ethics Committee of Taizhou Hospital of Zhejiang Province, China (K20240907) and was conducted in accordance with the principles of the Declaration of Helsinki.

### Immunohistochemistry

The TMAs were deparaffinized and rehydrated, and antigens were retrieved by microwaving in sodium citrate buffer (10 mmol/L, pH6.0). HLA-G expression was detected using immunohistochemistry (IHC). TMA slides were incubated with anti-HLA-G mAb 4H84 (1:500, Exbio, CZ) and then incubated with a horseradish peroxidase (HRP)-conjugated rabbit/mouse secondary antibody (1:100, Dako, Glostrup, Denmark). IHC staining was performed using a Dako EnVision kit (Dako, Glostrup, Denmark). HLA-G expression was imaged by using 3DHistech (Budapest, Hungary). IHC was performed as described in our previous study (29). The percentage of HLA-G expression was evaluated using 3DHistech and then reviewed by two blinded observers. The percentage of HLA-G-positive cells was calculated by cell counting but irrespective of staining intensity among the GC lesions and PTTs. A percentage of HLA-G of at least 5% was considered positive (30).

### Multiplex immunohistochemistry

In contrast to most previous studies reporting that HLA-G is absent in adjacent non-tumorous tissues or PTTs (7), our study observed that HLA-G expression was present in PTTs, which was further confirmed by Western blot with anti-HLA-G mAb 4H84 (39 KD, 1:1,000, Exbio, CZ) and anti-HLA-A,B,C,E mAb TP25.99SF (45 KD 1:1,000, Exbio, CZ) in six samples to discriminate the HLA-G expression from HLA-I antigens.

Moreover, our results showed that HLA-G expression was restricted to the fundic glands of the PTTs. However, the subpopulations of cells expressing HLA-G remain unclear. To define the HLA-G-positive subpopulation of cells in the gastric glands of PTTs and GC lesions, we performed multiplex immunohistochemistry (mIHC) staining on four PTTs (HLA-G positive by IHC) and two GC lesions (one HLA-G positive and one HLA-G negative by IHC). The gastric gland chief cell-specific marker PGA3/Pepsinogen I and mucous neck cell-specific marker MUC6/Mucin6 were used to identify the cell subpopulation expressing HLA-G by mIHC, in accordance with previous studies (31, 32).

mIHC staining was performed on 4-µm-thick, formalin-fixed, paraffin-embedded sections using a Bas Multiplex IHC Kit (Alpha X Beijing Biotech Co., Ltd., China). The staining procedure was performed according to the manufacturer’s protocol. Briefly, section slides were incubated at 60°C for 1 h, dewaxed, and hydrated, and antigens were retrieved using a Bas antigen retrieval solution (1:50, Alpha X Beijing Biotech Co., Ltd., China) in a microwave oven and blocked with 2% bovine serum albumin. Subsequently, the slides were incubated with the first primary anti-HLA-G mouse mAb 4H84 (1:200, 100 μL, Exbio, CZ) at 37°C for 1 h. They were washed three times with Tris-buffered saline with Tween 20 (TBST) and then incubated with the HRP-conjugated anti-rabbit/mouse secondary antibody (1:100, Dako, Glostrup, Denmark) at 37°C for 10 min. The slides were washed three times with TBST and incubated with a fluorophore solution (1:100, Bas Texas Red) in BAS signal amplification reagent (Alpha X Beijing Biotech Co., Ltd., China). The same procedure was repeated with the following primary antibodies: mouse mAb MUC6 (1:200, NBP2-44376, Novus Biologicals, USA) and rabbit Ab PGA3 (1:200, NBP2-54730, Novus Biologicals, USA). The fluorophore solution for mAb MUC6 was Bas Cy3 (1:100) and that for Ab PGA3 was Bas FITC (1:100). The slides were then incubated with the Bas DAPI working solution at room temperature for 5 min and finally with the mounting fluorescence quenching solution. The slides were imaged on a Zeiss Confocal Microscope LSM 800 with Airyscan (Carl Zeiss Microscopy, Germany), and the images were analyzed using ZEN (blue edition) software (Carl Zeiss Microscopy GmbH). The images were captured using the following emission/excitation wavelengths: 358/461 (DAPI), 588/616 (Texas Red), 494/525 (FITC), and 550/570 (Cy3).

### Statistical analysis

Chi-square test was performed to analyze the association of HLA-G expression status in PTTs and GC lesions with patient clinicopathological parameters. Kaplan–Meier method and log-rank test were performed to evaluate the relevance of PTTs and GC HLA-G expression status and clinicopathological parameters in the survival of patients with GC. Statistically significant parameters for survival were analyzed for hazard ratios (HR) using the Cox proportional hazards model. All statistical analyses and plotting were performed using IBM SPSS Statistics version 25 (IBM Corp., USA). Statistical significance was set at *p*<0.05.

## Results

### Clinical relevance of HLA-G expression in GC lesions and PTTs

**Figure 1:**
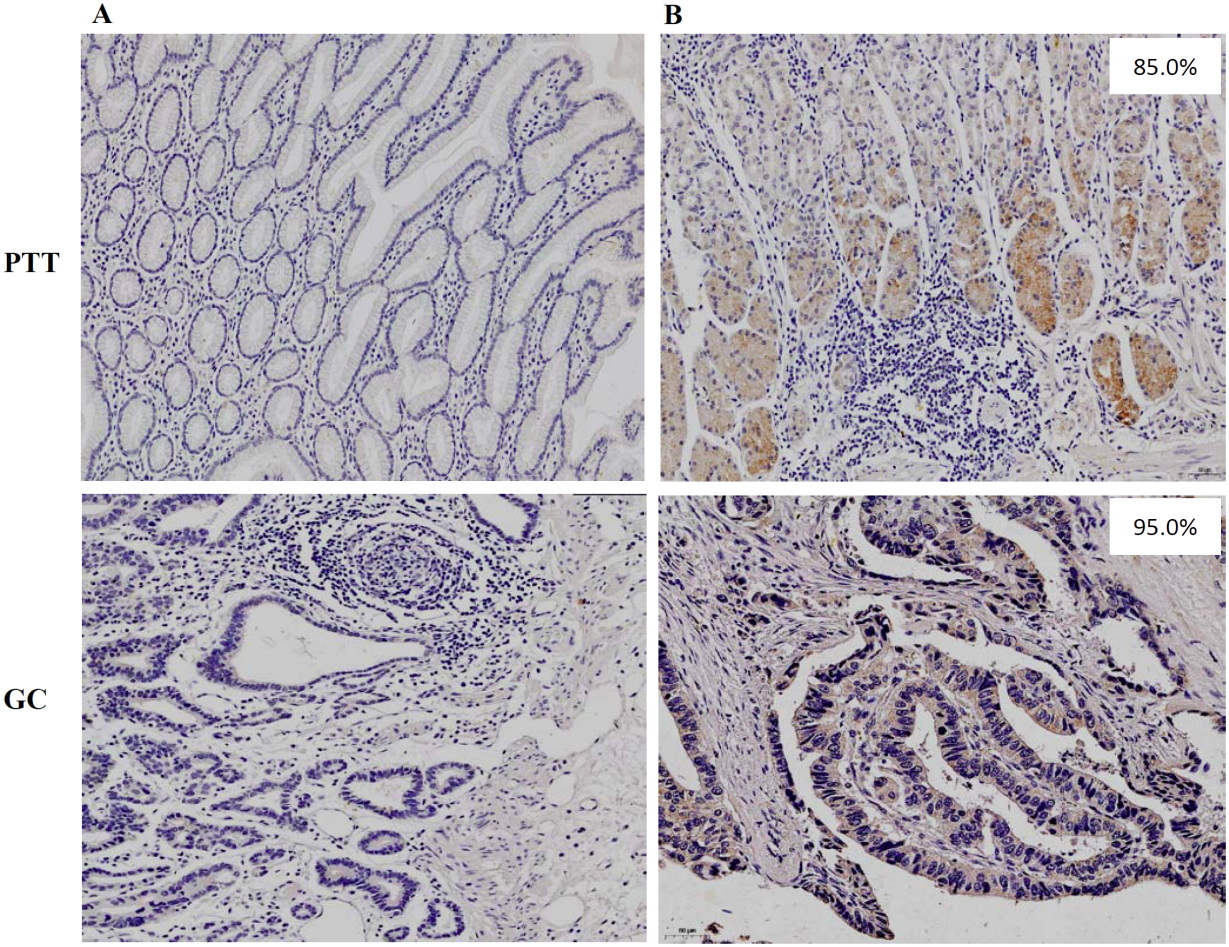
Immunohistochemistry staining of **(A)** negative HLA-G expression and **(B)** positive HLA-G expression in PTT and GC lesions, respectively (×100; mAb 4H84 1:500).

Both membrane and plasmatic HLA-G expressions were observed in GC lesions and PTTs, with variations in the positive percentages of HLA-G-expressing cells and staining intensity among samples. The percentages of HLA-G expression range from negative to 99%, with a mean of 56% for GC lesions (*n*=523) and from negative to 99%, with a mean of 66% for PTT tissues (*n*=283, *p*<0.001; **Supplementary Figure S1**). The HLA-G expression was further confirmed by Western blot in six PTT samples (**Supplementary Figure S2**), showing that mAb4H84 is specific for HLA-G detection. IHC staining also showed that HLA-G expression was restricted to the gastric glands in GC lesions and PTTs (**Figure 1**). Positive HLA-G expression was significantly more frequent in PTTs; positive HLA-G expression was observed in 70.7% (370/523) of GC lesions and 84.1% (238/283) of PTTs (*p*<0.001). Among the GC lesions, HLA-G expression was frequently observed in older patients (76.2% vs. 65.3%, *p*=0.006). Among PTTs, HLA-G expression was significantly associated with female sex (92.0% vs. 80.5%, *p*=0.014) and AJCC stages (*p*=0.043), whereas HLA-G expression was much higher in patients with GC of advanced AJCC stages (77.6% in AJCC_I+II_ vs. 87.4% in AJCC_III+IV_, *p*=0.031). No statistically significant association was observed between HLA-G expression status and pT, pN, and pM stages (**Table 1**).

**Table 1:** Relevance of HLA-G expression in GC lesion and PTT tissue with clinical variables.

Variables	GC lesion HLA-G				PTT HLA-G			
	Cases	Positive (%)	Negative	*p*	Cases	Positive (%)	Negative	*P* ^a^
Total	523	370 (70.7%)	153		283	238 (84.1%)	45	<0.001
Sex								
Male	353	252 (71.4%)	101	0.642	195	157 (80.5%)	38	0.014
Female	170	118 (69.4%)	52		88	81 (92.0%)	7	
Age								
≤63 years	262	171 (65.3%)	91	0.006	146	119 (81.5%)	27	0.218
>63 years	261	199 (76.2%)	62		137	119 (86.8%)	18	
Pt								
T_1_	26	18 (69.2%)	8	0.974	14	11 (78.6%)	3	0.509
T_2_	50	35 (70.0%)	15		24	18 (75.0%)	6	
T_3_	334	239 (71.6%)	95		197	169 (85.8%)	28	
T_4_	105	73 (69.5%)	32		45	37 (82.2%)	8	
pN								
N_0_	127	92 (72.4%)	35	0.959	61	50 (82.0%)	11	0.247
N_1_	81	58 (71.6%)	23		39	29 (74.4%)	10	
N_2_	136	96 (70.6%)	40		76	67 (88.2%)	9	
N_3_	178	124 (69.7%)	54		106	91 (85.8%)	15	
pM								
M_0_	498	351 (70.5%)	147	0.554	272	230 (84.6%)	42	0.293
M_1_	25	19 (76.0%)	6		11	8 (72.7%)	3	
AJCC								
I	49	37 (75.5%)	12	0.791	22	19 (86.4%)	3	0.043
II	148	104 (70.3%)	44		76	57 (75.0%)	19	
III	297	207 (69.7%)	90		172	152 (88.4%)	20	
IV	25	19 (76.0%)	6		11	8 (72.7%)	3	

a Comparison between or among each variable using the Pearson chi-square test.

### Prognostic significance of HLA-G expression in GC lesions and PTTs

**Figure 2:**
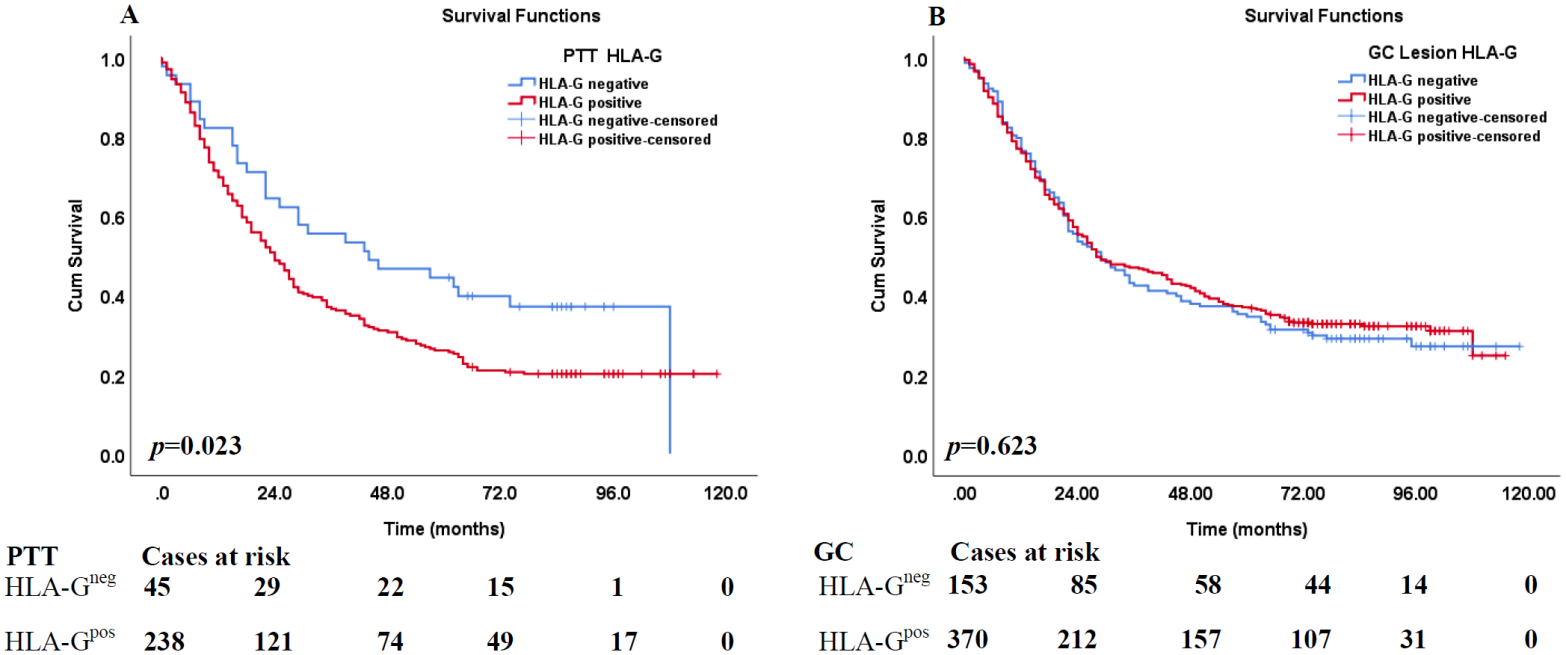
Kaplan–Meier analysis of HLA-G expression in PPT and GC lesions for the survival of GC patients. **(A)** Comparison of overall survival between HLA-G-negative and HLA-G-positive expression in PPT for CRC patients (*p*=0.023). **(B)** Comparison of overall survival between HLA-G -negative and HLA-G-positive expression in GC tumor lesions for CRC patients (*p*=0.523).

The prognostic significance of HLA-G expression status in GC lesions and PTTs and clinicopathological parameters in the survival of patients with GC was analyzed using Kaplan–Meier method and log-rank test. The results (**Table 2**) showed that the HLA-G expression status in PTTs was significantly associated with the survival of patients with GC, with those having HLA-G-negative PTTs demonstrating longer survival than those with HLA-G-positive PTTs (56.7 months vs. 42.0 months, *p*=0.023; **Figure 2A**). However, HLA-G-negative and HLA-G-positive status in GC lesions was not significantly associated with the survival of patients with GC (50.9 months vs. 52.1 months, *p*=0.623; **Figure 2B**). Among other clinical parameters, advanced clinical stages, including pT, pN, pM, and AJCC stages, were significantly associated with worse survival (all *p*<0.001). No statistically significant differences were found for sex (*p*=0.079) or age (*p*=0.081) in the survival of patients with GC.

**Table 2:** Log-rank analysis for the significance of the clinical variables in the survival of GC patients.

Variables	Group	Events/number	Survival (95% CI)	*p*
Sex	Male	246/374	55.0 (50.1–59.8)	0.079
	Female	135/182	46.8 (40.7–52.9)	
Age	≤63 years	189/287	55.8 (50.4–61.2)	0.081
	>63 years	192/269	48.8 (43.5–54.1)	
pT	T_1_	4/31	105.6 (98.6–112.5)	<0.0001
	T_2_	26/54	68.3 (58.2–78.4)	
	T_3_	265/355	44.7 (40.6–48.9)	
	T_4_	82/108	40.7 (33.1–48.3)	
pN	N_0_	42/135	89.7 (83.0–96.4)	<0.0001
	N_1_	50/83	60.8 (52.1–69.6)	
	N_2_	109/143	45.8 (38.8–52.7)	
	N_3_	179/194	25.4 (21.4–29.4)	
pM	M_0_	355/528	54.5 (50.5–58.5)	<0.0001
	M_1_	26/28	24.6 (13.1–36.2)	
AJCC	I	12/55	97.3 (88.9–105.6)	<0.0001
	II	75/153	69.8 (63.3–76.3)	
	III	266/316	34.8 (30.9–38.7)	
	IV	26/28	24.6 (13.1–36.2)	
PTT HLA-G	Negative	29/45	56.7 (44.1–69.4)	0.023
	Positive	189/238	42.0 (36.7–47.3)	
GC lesion HLA-G	Negative	109/153	50.9 (43.7–58.1)	0.623
	Positive	251/370	52.1 (47.5–56.8)	

Furthermore, among the 271 case-matched GC lesions and PTTs with available HLA-G expression status, similar results were obtained. HLA-G-negative status in PTTs was associated with significantly longer survival than HLA-G-positive status (57.7 months vs. 41.2 months, *p*=0.014). Moreover, HLA-G-negative and HLA-G-positive status in GC lesions, respectively, was not significantly associated with the survival of patients with GC (*p*=0.289; **Table 3**).

**Table 3:** Log-rank analysis for the significance of the clinical variables in the survival of case-matched GC lesion and PTT.

Variables	Group	Events/number	Survival (95% CI)	*p*
Sex	Male	143/187	45.1 (38.9–51.3)	0.567
	Female	68/84	40.9 (32.6–49.2)	
Age	≤63 years	104/136	47.2 (40.0–54.3)	0.194
	>63 years	107/135	39.1 (32.6–45.6)	
pT	T_1_	1/14	109.5 (102.8–116.1)	<0.001
	T_2_	16/23	52.6 (37.9–67.4)	
	T_3_	156/186	37.3 (32.2–47.4)	
	T_4_	36/45	36.0 (24.6–47.3)	
pN	N_0_	24/60	79.1 (68.1–90.1)	<0.001
	N_1_	29/39	49.0 (37.3–60.7)	
	N_2_	64/74	39.5 (31.3–47.7)	
	N_3_	94/98	21.3 (16.7–26.0)	
pM	M_0_	201/261	45.4 (40.2–50.6)	0.004
	M_1_	10/10	20.5 (1.0–40.0)	
AJCC	I	6/22	91.1 (75.1–107.0)	<0.001
	II	46/75	60.0 (50.9–69.2)	
	III	148/162	28.9 (24.3–33.5)	
	IV	10/10	20.5 (1.0–40.0)	
PTT HLA-G	Negative	28/44	57.7 (44.8–70.5)	0.014
	Positive	183/227	41.2 (35.8–46.5)	
GC lesion HLA-G	Negative	73/97	48.0 (39.3–56.7)	0.289
	Positive	138/174	41.1 (35.2–47.0)	

The aforementioned statistically significant factors were used to analyze the survival of the entire cohort of patients with GC, including sex, age, pT, pN, pM, and AJCC stages, and HLA-G expression status in PTTs using HRs derived from univariate and multivariate Cox proportional hazards models. Univariate Cox proportional hazards model results showed that both sex and age were not statistically significant factors for the survival of the GC patients. Multivariate Cox proportional hazards model results revealed that the HR for pT was 2.120 (95% confidence interval [CI]: 1.215–3.698, *p*=0.008), pN was 2.556 (95% CI: 1.102–5.929, *p*=0.029), pM was 1.799 (95% CI: 0.929–3.483, *p*=0.082), AJCC was 0.972 (95% CI: 0.403–2.343, *p*=0.949), and HLA-G expression status in PTTs of the entire cohort was 1.476 (95% CI: 0.984–2.213, *p*=0.060; **Table 4**).

**Table 4:** Cox proportional hazards analysis of variables for overall survival in the whole cohort of GC patients.

Variables	Categories	Univariate analysis		Multivariate analysis	
		HR (95% CI)	*p*	HR (95% CI)	*p*
Sex	Female vs. male	1.204 (0.976–1.486)	0.083	/	
Age	>63 years vs. ≤63 years	1.194 (0.976–1.460)	0.084	/	
pT	T_3+4_ vs. T_1+2_	3.359 (2.310–4.884)	<0.001	2.120 (1.215–3.698)	0.008
pN	N_2+3_ vs. N_0+1_	3.664 (2.888–4.649)	<0.001	2.556 (1.102–5.929)	0.029
pM	M_1_ vs. M_0_	2.333 (1.563–3.483)	<0.001	1.799 (0.929–3.483)	0.082
AJCC stage	III/IV vs. I/II	3.653 (2.866–4.655)	<0.001	0.972 (0.403–2.343)	0.949
HLA-G (PTT)	Positive vs. negative	1.561 (1.055–2.308)	0.026	1.476 (0.984–2.213)	0.060

HR, hazard ratios; 95% CI, 95% confidence interval.

Furthermore, among the 271 case-matched GC lesions and PTTs with available HLA-G expression status, the results were similar to those obtained for the entire cohort. The HR was 1.939 (*p*=0.022) for pT, 2.579 (*p*=0.024) for pN, 1.686 (*p*=0.024) for pM, and 1.048 (*p*=0.915) for AJCC, while the HLA-G expression status in PTT among case-matched cohort was 1.541 (95% CI: 1.023–2.321, *p*=0.039) for HLA-G expression status in PTTs (**Table 5**).

**Table 5:** Cox proportional hazards analysis of variables for overall survival in case-matched GC lesion and PTTs.

Variables	Categories	Univariate analysis		Multivariate analysis	
		HR (95% CI)	*p*	HR (95% CI)	*p*
Sex	Female vs. male	1.087 (0.814–1.451)	0.571	/	
Age	>63 years vs. ≤63 years	1.194 (0.911–1.565)	0.200	/	
pT	T_3+4_ vs. T_1+2_	3.014 (1.830–4.964)	<0.001	1.939 (1.099–3.420)	0.022
pN	N_2+3_ vs. N_0+1_	3.097 (2.257–4.249)	<0.001	2.579 (1.132–5.877)	0.024
pM	M_1_ vs. M_0_	2.474 (1.302–4.698)	0.006	1.686 (0.846–3.361)	0.137
AJCC stage	III/IV vs. I/II	3.212 (2.335–4.419)	0.001	1.048 (0.440–2.500)	0.915
HLA-G (PTT)	Pos vs. Neg	1.634 (1.097–2.435)	0.016	1.541 (1.023–2.321)	0.039

HR, hazard ratios; 95% CI, 95% confidence interval.

### Identification of HLA-G-expressing cell populations in the gastric glands

**Figure 3:**
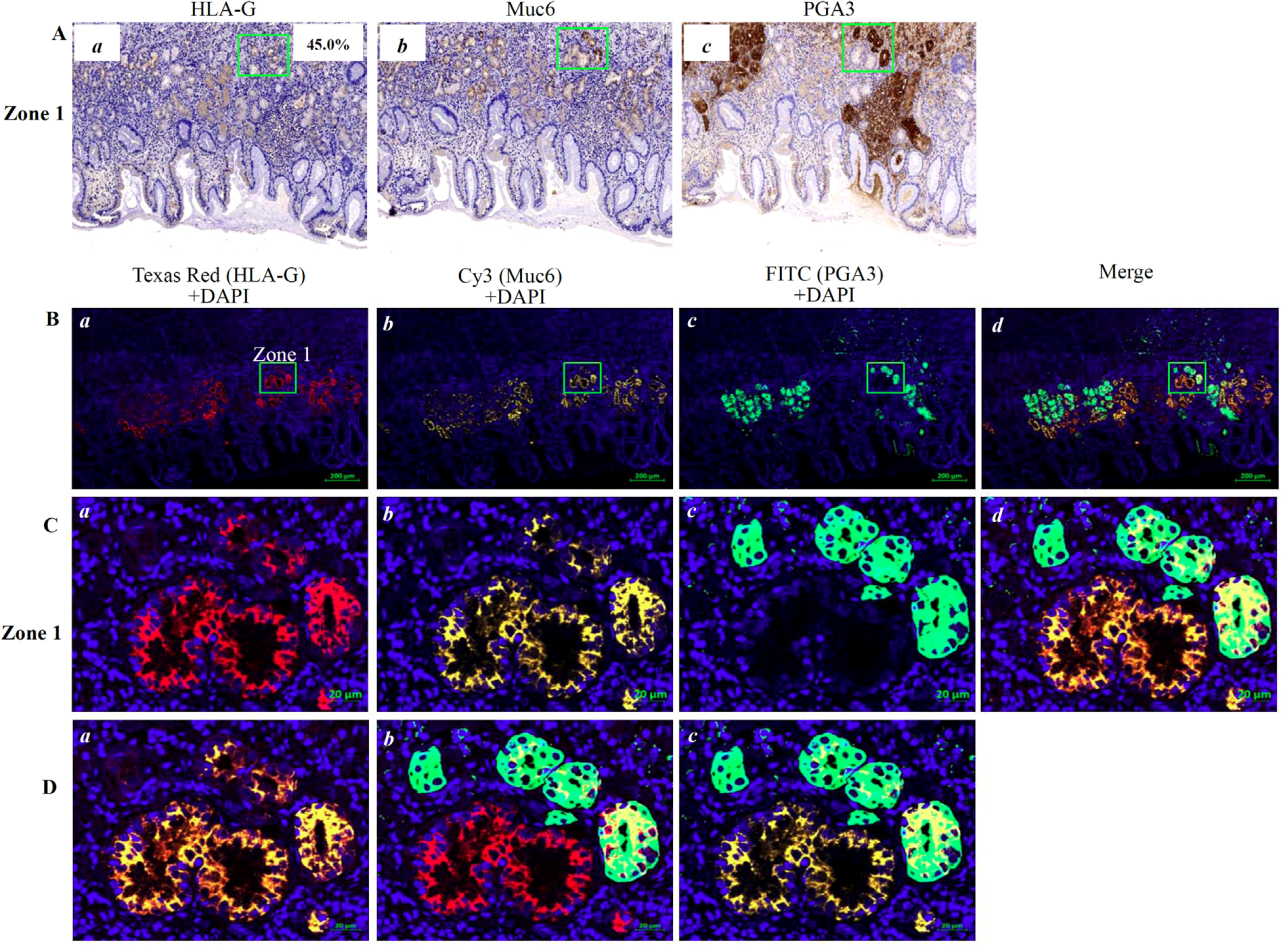
Immunostaining showing HLA-G expression in the cell population of the gastric gland in PTT. **(A)** Immunohistochemistry staining of **(a)** HLA-G, **(b)** MUC6 and **(c)** PGA3 cell populations of the gastric gland (×100). **(B)** Multiplex immunohistochemistry (mIHC) immunostaining showing the **(a)** HLA-G expression cells, **(b)** MUC6^+^ neck cell, and **(c)** PGA3^+^ chief cell in the gastric gland (bar=200 µm). The area in zone 1 was amplified and shown in **(C)** (bar=20 µm). **(D)** Images merged with **(a)** Texas Red/HLA-G+Cy3/Muc6+DAPI, **(b)** Texas Red/HLA-G+FITC/PGA3+DAPI, and **(c)** Texas Red/HLA-G+Cy3/Muc6+FITC/PGA3+DAPI.

**Figure 4:**
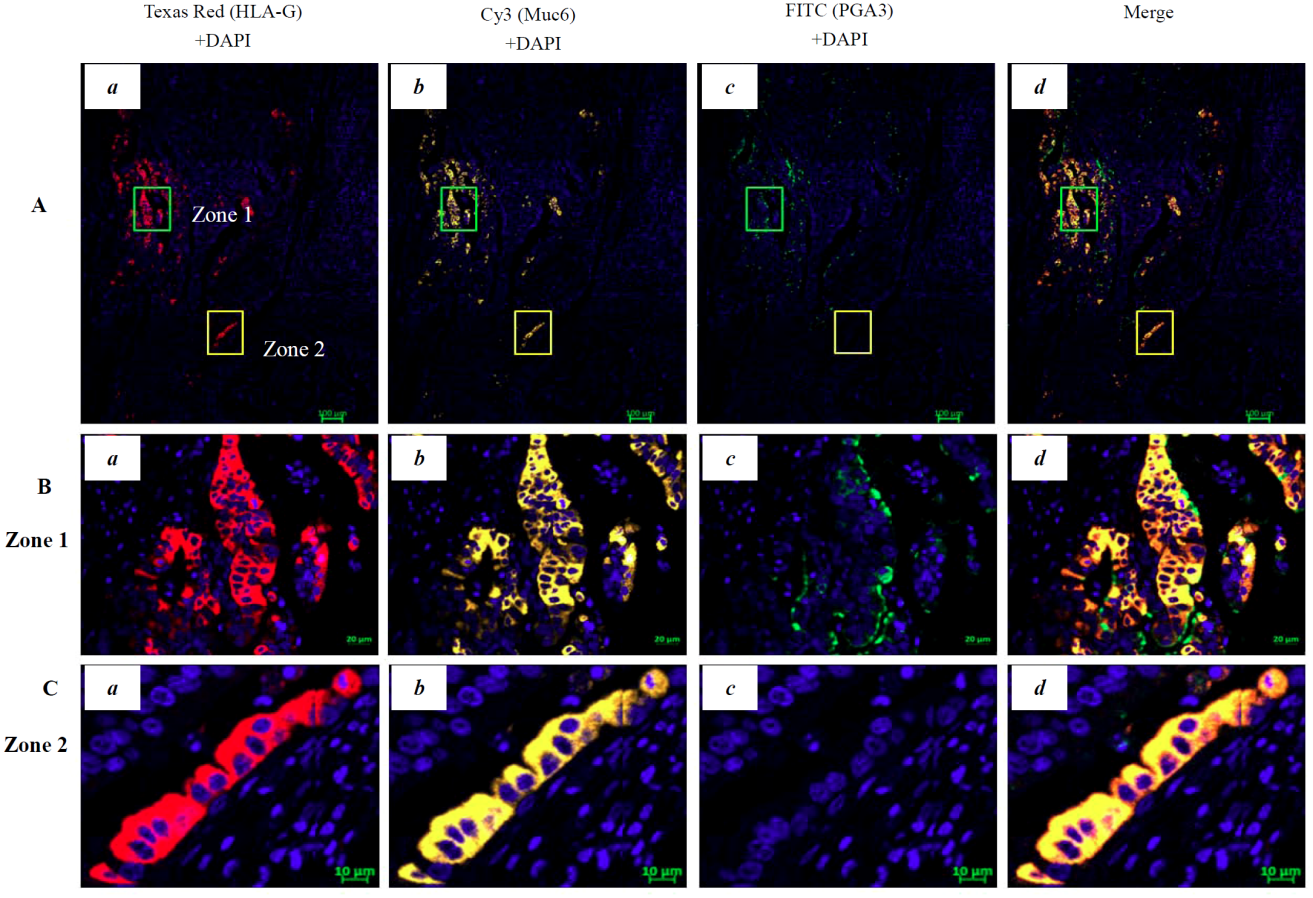
Multiplex immunohistochemistry (mIHC) immunostaining showing the HLA-G expression in the cell population of gastric gland in GC lesions. **(A)** Immunostaining showing the **(a)** HLA-G expression cells (Texas Red HLA-G+ +DAPI), **(b)** Cy3-Muc6+DAPI neck cell, **(c)** FITC-PGA3+DAPI chief cell, and **(d)** merged staining in GC lesions (Texas Red/HLA-G+Cy3/Muc6+FITC/PGA3+DAPI; bar=100 µm). The area in zone 1 and zone 2 was amplified and is shown in **(B)** (bar=20 µm) and **(C)** (bar=10 µm), respectively.

IHC staining showed that HLA-G expression was restricted to the gastric glands of PTT (**Figures 1B**, **3A-a**). We then used mIHC to identify the HLA-G-expressing cell subsets with the gastric gland chief cell-specific marker PGA3/Pepsinogen I and mucous neck cell-specific marker MUC6 as previously reported (31, 32). The mIHC results revealed that, in all four GC PTTs, HLA-G expression was restricted to MUC6^positive^PGA3^negative^ mucous neck cells, but negative in MUC6^positive/weak^PGA3^positive^chief cells (**Figures 3B-d, C-d, D-c** showed the merged staining). Similar results were observed in HLA-G-positive GC lesions: HLA-G expression was restricted to MUC6^positive^PGA3^negative/weak^ mucous neck cells (**Figures 4A-d, B-d, C-d** showed the merged staining).

## Discussion

Gastric cancer was the fifth most commonly diagnosed cancer (968,350 cases, 4.9% of the total cases) and the fifth leading cause of cancer-related death (659,853 cases, 6.8% of the total cancer-related deaths) worldwide in 2022 (33). Trends in the incidence (average annual percentage change [APCC] −2.9% for men and −2.8% for women) and mortality (APCC −3.7% for men and −4.3% for women) of GC were significantly decreased in 2000–2018. However, GC still ranked as the fifth most commonly diagnosed cancer (358,700 cases) and the third leading cause of cancer-related death (260,400 cases) in China in 2022 (34).

HLA-G expression was first observed in cytotrophoblasts in 1990 and in cancer in 1998 (1, 11). Different proportions and intensities of immune suppressor HLA-G expression have been frequently observed in various solid cancer lesions, while HLA-G is rarely expressed in tumor-adjacent non-tumorous tissues. An atlas of antigen-presenting-related genes generated from 4,323 publicly available RNA-Seq datasets by Boegel et al. (35) showed that the HLA-G transcript is expressed in various tissues. However, to date, proteomic HLA-G expression has only been observed in few cell populations in normal tissues, such as pancreatic islet β cells, erythroblasts, thymic medullary and subcapsular epithelium, tubule glandular epithelia, and glandular secretions in prostate gland and pituitary gland adenohypophyseal cells, testis Sertoli cells, and epidermal keratinocytes (3–6, 21, 36, 37). Upregulation of HLA-G expression can also be observed in macrophages in asthma with severe chronic inflammation, in mast cells in the liver and lung, and in kidney fibrosis (21, 38). In the context of cancers, several studies have shown that HLA-G expression in malignant lesions is associated with cancer progression and short patient survival, making it a promising target for cancer immunotherapy (17, 18, 20, 21, 39). HLA-G-targeted clinical trials for a wide range of advanced solid cancers with different strategies, including HLA-G antagonist alone (NCT04485013, NCT06380816) or combined with other immune checkpoint inhibitors and CAR-T (NCT05672459), are currently undergoing (https://clinicaltrials.gov/search?cond=HLA-G).

While most previous studies focused on the cancer itself, weak or absent HLA-G expression in PTTs has only been reported in a few studies with rather limited samples (6, 23). Glandular epithelia and/or secretions in normal prostate (*n*=5) and prostatic adenocarcinoma (*n*=4) were found to express HLA-G (6). Bai et al. (23) reported that normal (*n*=5) and cancer (*n*=97) endometrial tissues expressed HLA-G, while much higher levels of HLA-G expression were observed in endometrial cancer lesions. In this respect, HLA-G expression in PTT has rarely been explored; thus, whether HLA-G is expressed in PTT and its clinical importance remain largely unknown. PTTs have distinct molecular and biological behavioral features and clinical significance compared with their corresponding healthy tissues and cancer lesions (24–27). In this regard, peritumoral adipose-enriched stroma, but not myofibroblast-enriched stroma, was associated with poor survival of patients with breast carcinomas (26). Moreover, tumor-adjacent normal-tissue-derived transcriptomes were even better than those of primary tumors in predicting survival and disease recurrence in patients with colorectal cancer (27).

In patients with GC, previous studies have shown that tumor lesion HLA-G expression is significantly associated with advanced disease stage, susceptibility to immune suppression, disease progression, and poor patient survival (40–43). In contrast, in a study by Ishigami et al. (44), HLA-G expression in tumor lesions was markedly associated with better prognosis and prolonged survival in a cohort of 115 patients with GC. Chen et al. (45) reported that HLA-G expression was not significantly associated with worse survival in the entire cohort of 127 patients with GC but was significant in female patients. Although a meta-analysis revealed that HLA-G expression could be an independent prognostic factor for the poor survival of patients with GC, the divergent relevance of HLA-G in GC remains unclear (13). Among the aforementioned studies on HLA-G and GC, only two studies by Murdaca et al. (40) (number of PTT cases not described) and Du et al. (41) (*n*=179) had included PTT samples; however, no HLA-G expression was observed in non-neoplastic mucosa.

In this study, the clinical significance of HLA-G expression in GC lesions and PTTs was evaluated, and a specific HLA-G-positive subpopulation in PTTs and GC lesions was identified. Our results showed that HLA-G expression was more frequently observed in PTTs than in GC lesions (84.1% vs. 70.7%, *p*<0.001) and that HLA-G expression in PTTs (HR=1.561, *p*=0.026), but not in GC lesions, was associated with poor survival of GC patients. Our results agreed with previous studies that HLA-G expression status was not associated with worse prognosis (12, 45, 46). Disparities in the prognostic significance of HLA-G expression in patients with GC were multifactorial, such as high intratumor and intertumor heterogeneous expression of HLA-G, and variations in methodological protocols to determine the HLA-G expression among these studies were noted (12, 39).

We found for the first time that MUC6-positive fundic gland mucous neck cells in PTT were the predominant subpopulation expressing HLA-G. PTTs characterize a distinct microarchitecture and microenvironment that can facilitate tumor progression through a cross-talk between mechanical signaling and immune activity. Moreover, its informative importance in cancer tumorigenesis, metastasis, prognosis, drug response, and disease recurrence has been highlighted in various types of tumors (47–50). In line with this, differential gene expression in normal breast epithelium in PTT was reported to reflect the differences of ER^+^ and ER^-^ status in invasive breast cancer, which could help identify early genomic events in breast cancer development (50). Hippo-related gene expression in PTTs can predict the prognosis of patients with hepatocellular carcinoma. In addition, transcriptomes and immune cell composition in PTT are much better than those in primary tumors for survival and tumor recurrence prediction among patients with CRC (27, 51).

PTT is an intermediate phase between tumors and healthy normal tissues. Histologically, normal PTT has distinct molecular characteristics compared with healthy normal tissue but shares transcriptional similarity with its corresponding tumor to a certain degree (52). A stepwise alternation of molecular and transcriptional accumulation in PTT is critical for cancer tumor development. TIGIT^+^CD20^+^ B cells in the PTTs of GC are an independent prognostic predictor of worse survival among patients with GC (53). In this scenario, HLA-G expression in fundic gland mucous neck cells in PTTs could play immunological modulation and pro-tumorigenic roles in pre-cancerous malignant transformation. The chief cells, which originate from mucous neck cells, are involved in tumorigenesis, which could serve as a source of GC cells *via* stem/progenitor cells (54, 55). Fundic gland chief cell-predominant type gastric adenocarcinoma (MUC6^positive/weak^PGA3^positive^) is a rare variant of well-differentiated adenocarcinoma and a novel disease entity (56). Inflammation, familial and environmental factors such as *Helicobacter pylori* infection, smoking, and irradiation are considered risk factors for GC (55, 57). In this regard, several studies reported genetic variations of HLA-G related to the susceptibility of *H. pylori* infection and GC progression and prognosis (58–61). Moreover, HLA-G expression was found to be associated with *H. pylori* infection and inflammation in gastric antrum (62).

However, our study obviously has limitations. First is the sample size in multiplex IHC to localize the HLA-G expressing cell subsets in the fundic gland, where only four PTTs and two GC lesions were performed; more samples are critically necessary to solidify our preliminary results that HLA-G expression is restricted to gastric mucous neck cells. Secondly, though HLA-G expression in PTTs is significantly related to the poor survival of GC patients, HLA-G in PTTs of GC patients are not an independent prognosticator when adjusted with other confounders including sex, age, and the patient’s clinical disease stage.

In summary, our preliminary findings revealed, for the first time, that HLA-G expression is restricted to fundic gland mucous neck cells and that HLA-G expression in PTTs is significantly related to the poor survival of patients with gastric cancer. However, underlying mechanisms, potential biological roles, and clinical significance of HLA-G expression in mucous neck cells in gastric cancer development remain unknown and require further exploration.

## Data Availability

The original contributions presented in the study are included in the article/**Supplementary Material**. Further inquiries can be directed to the corresponding authors.

## Supplementary material

Supplementary Figure 1 Distribution of the percentage of HLA-G^+^ cells in gastric cancer lesions and PPTs.

Supplementary Figure 2 Determination of HLA-G expression in PTTs with Western blot. PTT lysates (P1–P6) were probed **(A)** with anti-HLA-ABCE mAb TP25.99SF (1:1,000, Exbio) and **(B)** with anti-HLA-G mAb4H84 (1:1,000, Exbio).
