# Supplementary figures and images for: HLA-G expression in peritumoral fundic gland mucous neck cells, but not in tumor lesions, related to poor survival in patients with gastric cancer
Source: Front Immunol. 2025 Oct 16;16:1660054. doi: 10.3389/fimmu.2025.1660054 (PMC12571866; doi:10.3389/fimmu.2025.1660054)

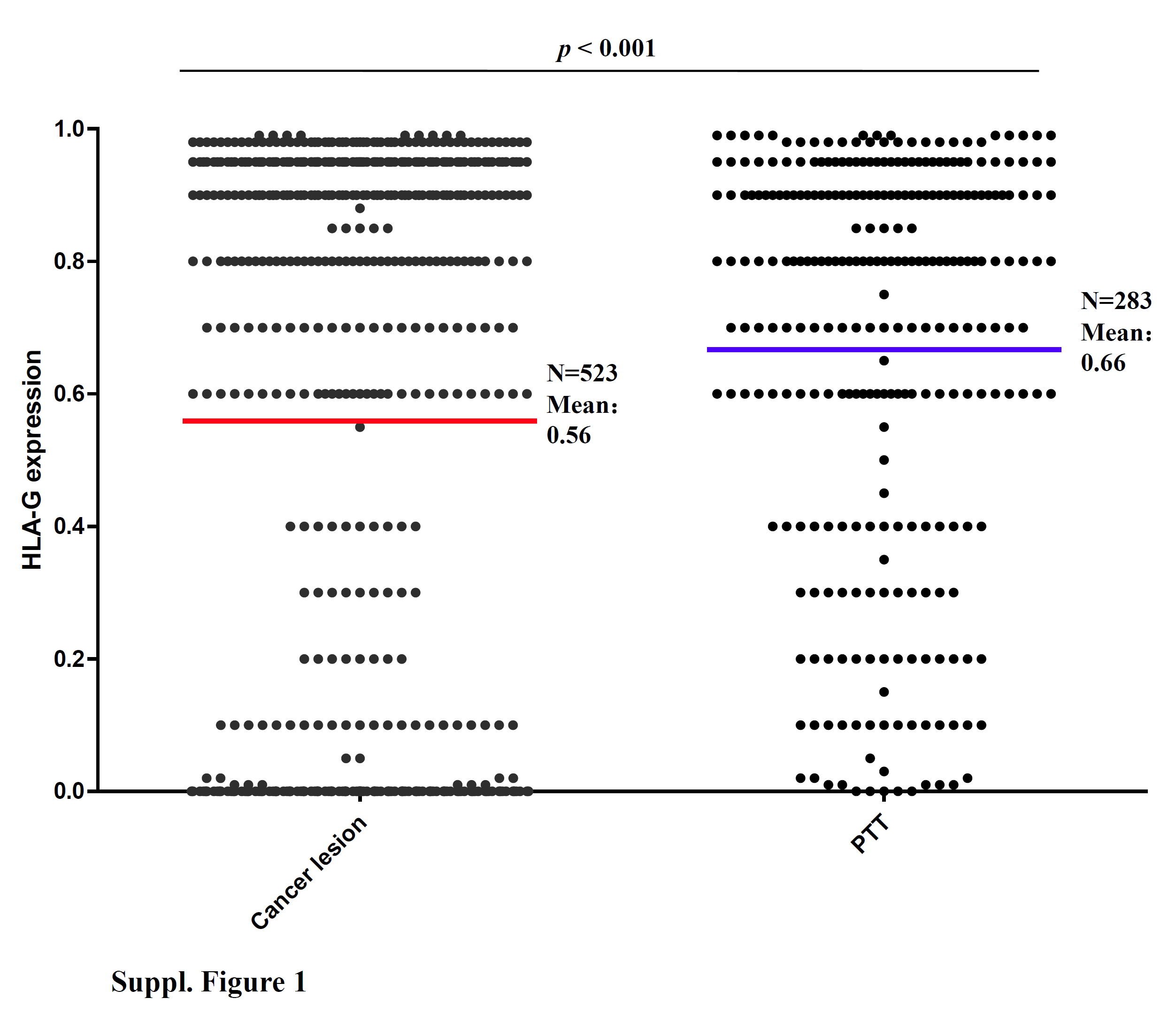

Supplement: Supplementary Figure 1 — Distribution of the percentage of HLA-G+ cells in gastric cancer lesions and PPTs. [file Image1.tif]

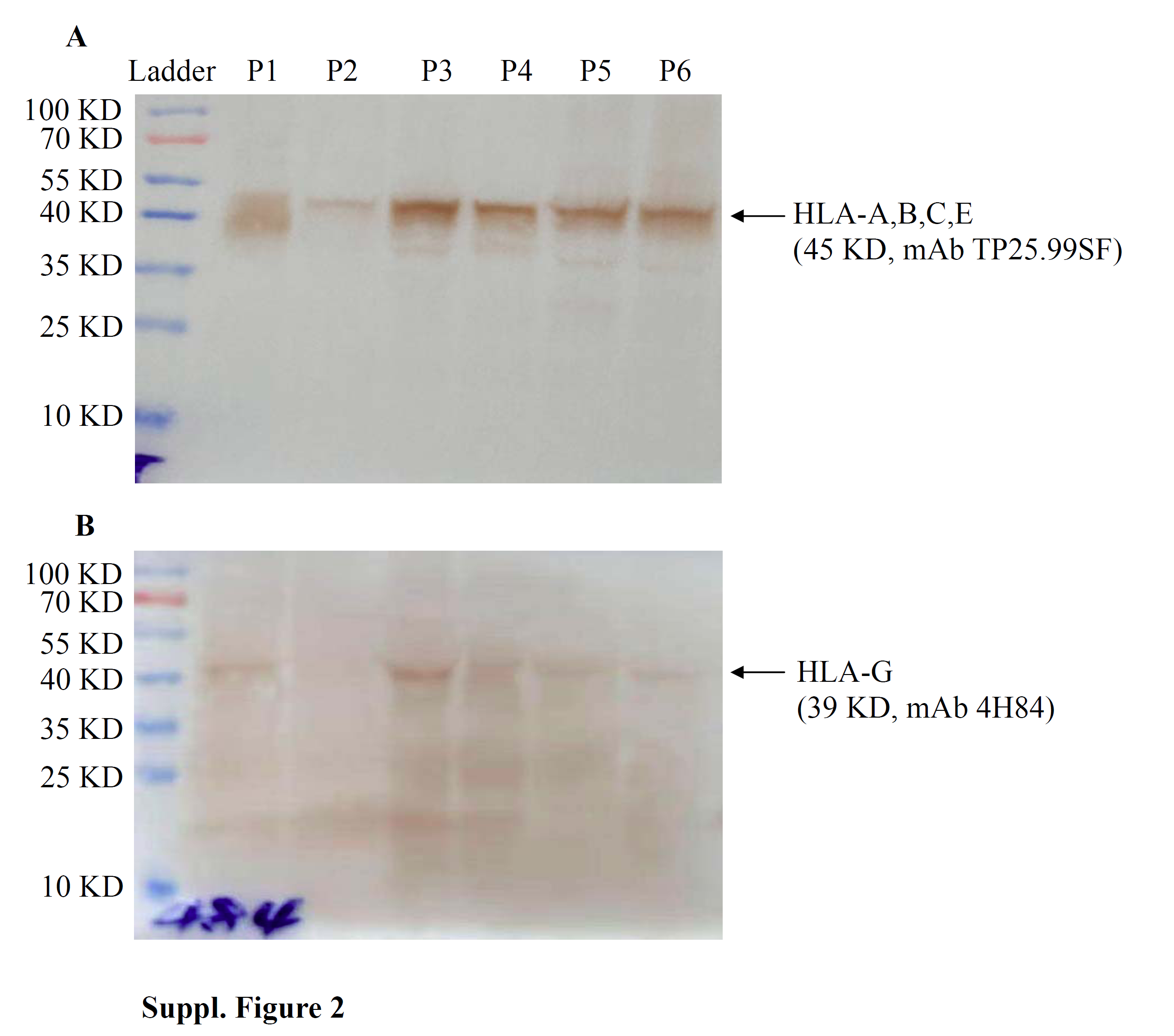

Supplement: Supplementary Figure 2 — Determination of HLA-G expression in PTTs with Western blot. PTT lysates (P1–P6) were probed (A) with anti-HLA-ABCE mAb TP25.99SF (1:1,000, Exbio) and (B) with anti-HLA-G mAb4H84 (1:1,000, Exbio). [file Image2.tif]
